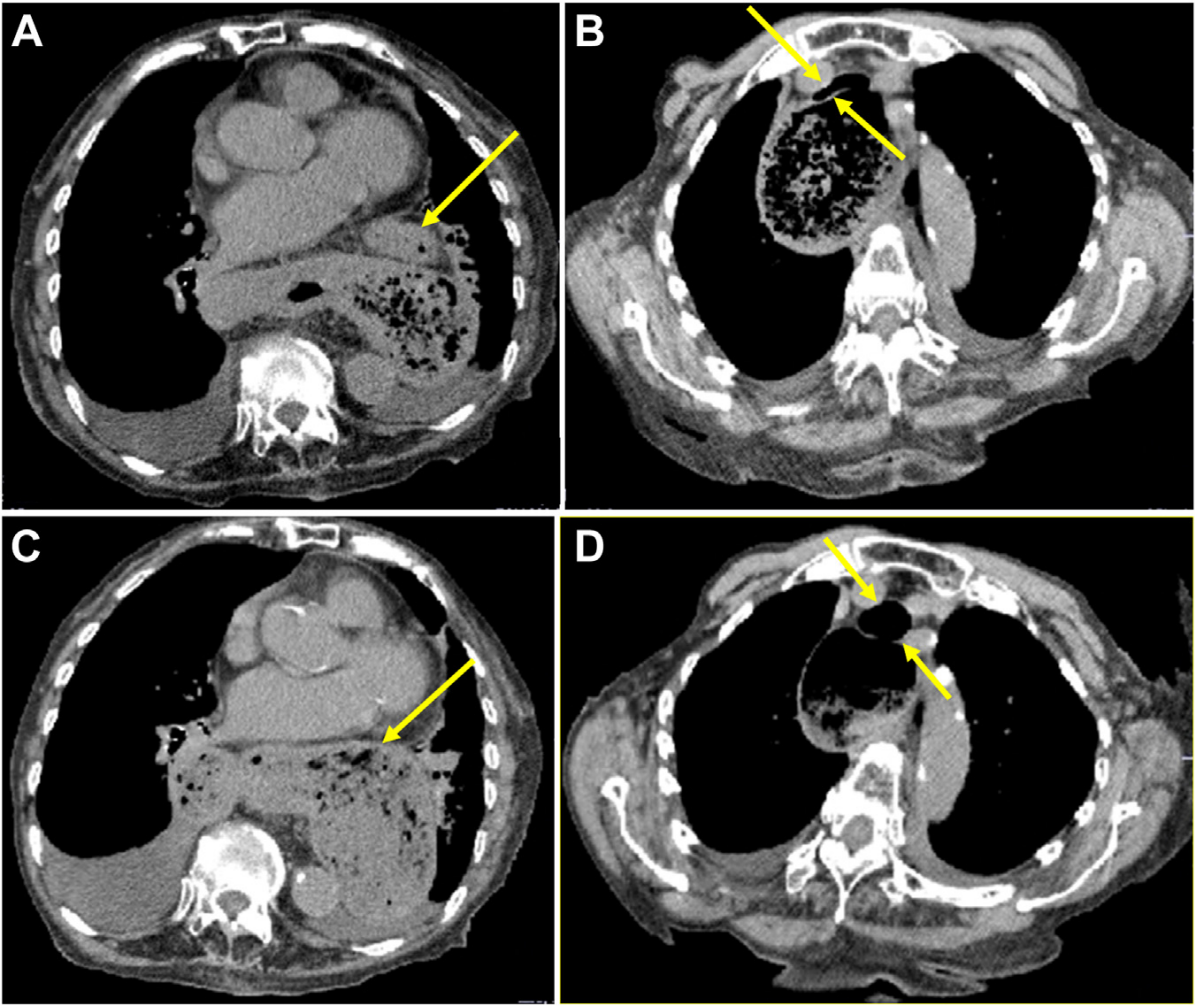# Recurrent Respiratory Distress Caused by Temporary Herniation of Transverse Colon Into Hiatal Hernia in Patient With Kyphosis

**DOI:** 10.1016/j.gastha.2024.10.009

**Published:** 2024-10-16

**Authors:** Hideaki Kazumori

**Affiliations:** Department of Gastroenterology, Matsue Seikyo General Hospital, Matsue, Shimane, Japan

A 97-year-old woman with thoracic kyphosis was brought to our hospital on several occasions with respiratory distress, though the cause could not be clarified for the long term. The ambulance crew noted respiratory status based on effort ventilation with oxygen saturation of 50%, with the respiratory condition relieved soon after arrival.

Following another respiratory distress incident 1 month later, CT findings showed entry of both the transverse colon and entire stomach into the thoracic cavity (Figure *A*, arrow: colon). The compression of the stomach by the transverse colon had caused obstruction to the passage of the esophagus. As a result, the esophagus was extremely dilated, which was followed by tracheal compression (Figure *B*, arrows: trachea). After resolution of respiratory symptoms, CT indicated that the transverse colon had exited the thoracic cavity, with no compression of the stomach (Figure *C*, arrow: stomach) or trachea observed (Figure *D*, arrows: trachea). Thus, respiratory distress caused by herniation of the transverse colon was confirmed. An effect of kyphosis was suggested to be increased abdominal pressure, resulting in creeping of the transverse colon into the thoracic cavity.

When a hiatal hernia involves the transverse colon, intrusion into the thoracic cavity usually remains. Presented here is an extremely rare case, with movements into and out of the thoracic cavity easily repeated making it difficult to determine the pathogenesis.

**Figure:**